# Progress in Heart Surgery: Let the Magic Continue

**DOI:** 10.3389/fsurg.2014.00033

**Published:** 2014-08-29

**Authors:** Hendrik T. Tevaearai Stahel

**Affiliations:** ^1^Clinic for Cardiovascular Surgery Inselspital, Bern University Hospital, University of Bern, Bern, Switzerland

**Keywords:** heart surgery, cardiac surgery, translational research, new developments, challenges, management

At the age of 7, I required an urgent operation for a broken, awfully deformed wrist. It was the first day of “summer” vacation in Tahiti where I lived, which made it even more painful. On the other hand, it became a revelation as I was offered the restricted opportunity to enter an operating room. Despite premedication and blurred vision, I clearly remember this surreptitious atmosphere of the OR, the place where magic happens. I wanted to become a magician; I decided to be a surgeon.

Years have passed and medicine has advanced. Magnificently, if one considers the numerous new drugs and devices that have been developed, as well as the various technologies such as those now routinely used in imaging and surgical facilities. Heart surgery is certainly a good example to illustrate this splendid progression, as many approaches regularly performed nowadays were simply not imaginable only a couple of decades ago. Importantly, not only have the techniques changed, the results have greatly improved as well.

## So What Can We Do Now?

Have we reached our limit? Can we not do better than what some of us already consider as good enough? And even if we can perform better, do we want this and, simply, can we afford it?

These are legitimate questions, which preoccupy essentially politicians and insurance providers, although it should be us, cardiac surgeons, who take control of our future, or at least provide our political, administrative, industrial, and regulatory partners with reliable facts regarding the current situation and possible clinical, scientific, economical, and developmental perspectives. Since we are not alone in the decision making process, it is indeed our responsibility to assist our partners to the best of our abilities, in order to safeguard all of the thrilling aspects that make our profession attractive and promising, or to be more explicitly, having access to the best technologies for our patients, providing the best training for our students, and making the best out of our scientific ideas. We must be convincing, and we must be heard.

The specialty section Heart Surgery in *Frontiers in Surgery* is an ideal platform for this. It will obviously incorporate all topics in the field of cardiac surgery, with a specific emphasis on relevant R&D advances and reliably performed clinical studies. But we also aim to provide an interactive platform for the exploration and discussion around the limits of heart surgery, to go one step beyond these frontiers and build our future. *Frontiers in Surgery* with its section Heart Surgery is therefore more than just an additional journal by and for heart surgeons. It is a journal that integrates opinions of all partners working alongside heart surgeons to constantly improve quality in heart surgery.

Let us face it; the good results of today’s cardiac surgery cannot be attributed to a mere handful of surgeons, even if very good and highly publicized. It is rather, as it has always been, due to the integrated and coordinated forces of dedicated groups of clinical partners, teams of surgeons, perfusionists, anesthetists, and intensivists, very well trained nursing staff, physiotherapists, and rehabilitation specialists, as well as cardiologists and general practitioners. Thus, the greatest innovations in cardiac surgery result from this shared vision and collaborative accomplishment. And this mindset must continue. In fact, we must constantly reevaluate and make an effort to understand the contributions and needs of our partners. Three of these groups, with whom our interactions represent both ongoing and upcoming challenges, must be considered very seriously, namely, (1) the new generation of doctors, some of whom will be our successors, (2) the scientists who provide us with more and more sophisticated biological and clinical tools that must be integrated into our practice, and (3) the industrial and the regulatory partners, who take more and more independence regarding the development, evaluation, and marketing of new technologies and drugs.

## Does the New Generation Qualify to Take Over?

Whereas we were taught to work hard and long hours in order to attain a solid training and reliable autonomy, the younger generation clearly professes other priorities and is definitely no longer willing to forgo values like family and life balance. Today, part-time clinical duties are not only a demand of our women colleagues but are also slowly becoming reality in surgical units among both sexes and across all positions. In fact, more and more women successfully complete cardiac surgery training programs, leading to a more balanced ratio of female:male surgeons. In many countries, working rules/laws have also become more rigorous and force us to imagine other models to guarantee quality patient care. This may be criticized, but we can also consider it as an opportunity to rethink our model.

Whereas we basically lived together, learned from each other, and were all always informed about every aspect of our clinic, perhaps we must start learning to communicate differently, delegate more, for example, to dedicated internists who could run our ward, and focus our energy more on other specific aspects of our job in which we excel. This change would, however, implicate a new, corporate way of thinking, a new training mindset – in other words a new culture. And it also would require new structures with coordinators/managers in order to reach another level of stability. Is it really impossible? These are major changes, yes. But other fields have undergone similar restructuration, and no one questions the fact that pilots are not allowed to fly more than 30 h in 7 consecutive days, for example.

## What Can We Learn from Our Laboratory and Clinical Scientists?

The rapid development in biological and material technologies makes it difficult for clinicians, including heart surgeons, to keep track of the true implications or promises for daily activities. Following the age of gene and cell therapy, it seems that we are now entering a new era of personalized medicine. However, even though it is slowly becoming evident that each individual may respond slightly differently to one specific therapy, the bases for understanding these differences rely on highly specific knowledge of the interaction between genomics, proteomics, metabolomics, and environment, and will thus probably remain obscure for most of us. In other words, learning from highly specialized new fields in medicine may simply become an impossible task and instead we might be forced just to trust it.

But we can still learn at a more traditional level of research and development. And one would actually expect that learning nowadays be largely facilitated by the relatively straightforward access to a very large amount of information. For instance, typing “cardiac surgery” in Pubmed provides 16,000 hits for the year 2013, whereas it was 6,000 less only a decade ago, and the evolution over these last years does not show any evidence of reaching a plateau. Do we really have so much more to publish these days than we did before? Or is this representative of a win–win mentality that is slowly gaining ground between authors, who are still pushed to publish, and publishers who use this opportunity to expand their activity? Publishing was originally aimed at informing peers about results that could potentially help the community to achieve better outcomes. But too many reports make it difficult to catch the most relevant and the most appropriate information. In addition to this phenomenon, an increasing number of frauds and retractions have also been reported, contributing to sow confusion in the medical community. New ways of guarantying the quality of studies and publications are thus mandatory, and may include the extension of monitoring and auditing activities as well as certification of research teams. Obligatory ethical approval for every single study, including retrospective and quality oriented analyses, will likely contribute to increasing the overall quality of reports and studies. Several groups have also started assembling their results in national or international registries, having thus the opportunity for more precise and detailed analyses. However, the value of large amounts of compiled data is realized only when these registries are representative of the true situation and include either all patients from a region or a country, such a national registry, or at least a representative sample of this population. This necessitates a data management structure with dedicated personnel, certified material, and standard operating procedures regarding data processing (data definitions, collection, checking, completing, transfer, and archiving), data monitoring, and independent data analysis (integrity and transparency). In other words, it necessitates a true professionalization of research units, which represents a critical investment that is, effectively, not realistic for every individual center.

## Shall We Rely on Industrial Partners and Authorities to Take Control of the Development of Our Future Technologies?

There was a time when pioneers used their laboratories as true platforms for direct translational development. Problems faced in daily activities were mocked-up *in vitro* or *in vivo* and solutions were rapidly transferred back to the clinic. This relatively uncomplicated art of technology transfer, even though unimaginable nowadays, truly allowed the rapid development of cardiac surgery and cardiology, and subsequently the spectacular improvement, in less than half of a century, of survival of patients with cardiac diseases. Things have radically changed and today ideas are rarely based on needs originally identified by clinicians, and clinicians rarely undertake the development of their own ideas. It is a fact that the pathway from an idea to a registered and commercialized product has become, over the years, a complex, lengthy, and expensive process. But do we want our role to be restricted; or do we want to take back our roles as innovators and creative leaders, and can we still do it?

In fact, one must first of all not forget that clinicians still play a critical role in product development. Some of us indeed act as consultants for medical companies or investigators in clinical trials. Nevertheless, the overall duration of new product development until market authorization has drastically increased in length and complexity over the last two decades, especially for drug development, and a series of specialized intermediary partners are now needed to achieve this goal. In parallel, the investment required for such development has become colossal, and the risk that the product will not be granted market authorization remains high throughout development. Consequently, companies tend to limit their development pipeline to projects with significant chances of success, whereas other ideas may be abandoned early. As clinicians, however, we remain those who face the patients and we certainly do not want to accept a reality dictated primarily by economic interests. We therefore must be more active in the development process, learn the basic aspects related to translational research pathways and integrate them into our training curriculum and/or post-graduate education. We need to take advantage of opportunities, already existent in many countries, to follow health management and medical technology courses, which should, with time, contribute to refocusing our way of thinking onto our real needs and how to realize them.

## Conclusion

Cardiac surgery is only a few decades old, but seems to have already reached maturity. After a fantastic beginning, cardiac surgery is currently undergoing a stable period, in which results are good and patients seem happy. But although no big revolution is anticipated in the coming years, we can still do better. For this, we must encourage ourselves and our current and future collaborators, our peer doctors, our scientists, our industrial partners, and our legal, ethical, and regulatory partners to collaborate in a new endeavor. Together, we can strive to learn more, better, and faster in the cardiosurgical field and to make this knowledge available to our patients.

## Conflict of Interest Statement

The author declares that the research was conducted in the absence of any commercial or financial relationships that could be construed as a potential conflict of interest.

